# Sex-specific short- and long-term outcomes in patients with leadless cardiac pacemakers

**DOI:** 10.1007/s00392-026-02891-w

**Published:** 2026-03-17

**Authors:** Daniel Kiblboeck, Karim Saleh, Jakob Boetscher, Hannah Rohringer, Julian Maier, Justin Lacher, Christian Reiter, Joerg Kellermair, Helga Wagner, Stefan Raidl, Thomas Lambert, Clemens Steinwender, Hermann Blessberger

**Affiliations:** 1 Department of Cardiology and Medical Intensive Care, Kepler University Hospital, Medical Faculty, Johannes Kepler University, Altenbergerstrasse 69, Linz, 4040 Austria; 2 Clinical Research Institute for Cardiovascular and Metabolic Diseases, Medical Faculty, Johannes Kepler University, Linz, Austria; 3 Center for Clinical Studies, Johannes Kepler University, Linz, Austria; 4 Department of Applied Statistics, Medical Statistics and Biometry, Johannes Kepler University, Linz, Austria; 5 Department of Internal Medicine II, Paracelsus Medical University, Salzburg, Austria

**Keywords:** Leadless cardiac pacemaker, Sex-specific disparities, Indications, Complications, Outcome

## Abstract

**Background:**

Safety and efficacy have been well demonstrated for Micra™ leadless cardiac pacemakers (LCPs). However, the presence of sex-specific disparities remains unclear.

**Methods:**

The aim of this single-centre observational study was to assess the sex-specific short- and long-term outcomes in patients undergoing LCP implantation.

**Results:**

In total, 378 LCPs were implanted in 127 women (33.6%) and 251 men (66.4%). The most frequent indications included atrial fibrillation with slow conduction (women: 31.5%, men: 44.6%), third-degree atrioventricular block (women: 31.5%, men: 33.5%) and sick sinus syndrome (women: 21.3%, men: 9.6%). Electrical performance parameters of LCPs were similar between sexes. Procedure-related complications during LCP implantation occurred more frequently in women (3.1%) compared to men (0.4%), though no difference was observed for all complications during the index stay (women: 3.9%, men: 1.6%, *p* = *0.18*). In-hospital mortality was low for women (0.8%) and men (0.8%, *p* = *0.96*). A multivariable logistic regression analysis adjusted for sex, age, diabetes, chronic kidney disease, coronary artery disease and transcatheter and surgical valve replacement revealed concomitant lead extraction (OR 9.153, *p* = *0.001)* as the only predictor for complication or death during index stay. All-cause mortality was 30.7% in women (*n* = 39) and 27.5% in men (*n* = 69, *p* = *0.28)* during a median follow-up of 41 months (IQR 22–65 months).

**Conclusions:**

No sex-specific disparities were observed with respect to complications during index stay, in-hospital and all-cause mortality. Less frequent use of LCP therapy in women may relate to differing indications between sexes. Further prospective studies may help to develop sex-specific recommendations for LCP therapy.

**Graphical Abstract:**

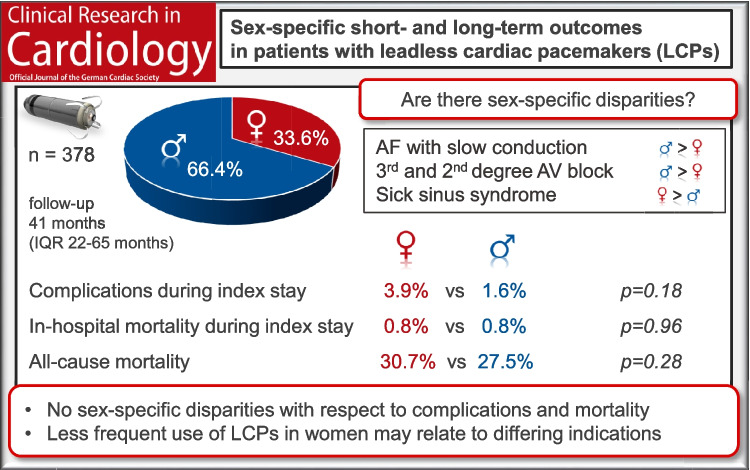

## Introduction

Leadless cardiac pacemakers (LCPs) represent an innovative approach to managing bradyarrhythmias, offering advantages over traditional transvenous pacemakers (PMs) [1]. By eliminating the need for leads, LCPs reduce potential complications such as lead dysfunctions, infections, and pocket hematomas. Despite these benefits, concerns remain regarding how anatomical and physiological differences between sexes may influence the safety and outcomes of LCP therapy.

Sex-based disparities in outcomes have been reported with transvenous PMs, with women often experiencing higher complication rates [2–4]. Potential contributing factors include sex-specific anatomical differences such as smaller heart size and vascular access diameter as well as physiological variations and differences in pacing indications. These differences may have important implications in the context of LCPs, which rely on intracardiac fixation and precise device placement.

A study by Huang et al. reported similar outcomes for both sexes after Micra™ LCP implantation, with low device-related complication rates but relatively high 30-day all-cause mortality rates of 6.7% in women and 6.8% in men [5]. Recently, a large United States database study revealed a lower utilisation of LCPs in women, along with higher rates of co-morbidities and major complications compared to men, as well as elevated in-hospital mortality (women: 5.5%, men: 4.5%, *p* < 0.01) [6]. These findings prompt critical questions: what drives the less frequent use of LCPs in women, and what sex-specific factors contribute to higher complication and mortality rates?

The rationale of this study was, to assess whether women undergoing LCP implantation experience higher complication and mortality rates, which might contribute to a less frequent use of LCP in women. Therefore, the study aimed to investigate sex-specific disparities in pacing indications, procedural outcomes, complications, device performance parameters and both short- and long-term outcomes at a high-volume LCP implantation centre in Austria.

## Methods

### Study design and outcomes

A retrospective analysis was conducted using the local LCP registry at our institution, including patients who underwent Micra™ LCP (Medtronic, Minneapolis, MN, USA) implantation between December 2013 and October 2022. During the study period no other LCP systems were implanted at our centre.

Patients were scheduled for LCP implantation according to current European Society of Cardiology (ESC) Guidelines on cardiac pacing and a national expert consensus document of the Austrian Society of Cardiology [7, 8]: Good candidates for LCP implantation were patients with no need for AV sequential pacing, CRT or transvenous ICD, history of CIED infection, permanent AF and AV block or slow ventricular response, missing or difficult venous subclavian access, history of complications of PM therapy, elevated risk of complications or risk of tricuspid valve dysfunction. Possible candidates for LCP implantation were patients suitable for a LCP according to underlying arrhythmia, two or more risk factors for device infection (diabetes mellitus, renal dysfunction or chronic hemodialysis, chronic use of corticosteroids, recurrent systemic infections or immunosuppressive therapy), transient sinus arrest or AV block with need of backup pacing and very low anticipated ventricular pacing rate (< 1–5% of beats). Possible candidates for LCP implantation under certain circumstances (e.g. older age and co-morbidities) were patients with transient or permanent AV block or SSS with transient or permanent bradycardia with anticipated ventricular pacing rate exceeding 5% of beats, recurrent syncope due to vagally induced cardio-inhibition (sinus bradycardia or transient AV block), frequent sports activity stressing the shoulders (golf, hunting, etc.), age < 65 years, children and adolescents < 20 years of age. No candidates for LCP implantation were patients with expected high ventricular pacing burden and LV dysfunction (LVEF ≤ 50%).

Results were stratified by sex. The primary objectives included the assessment of sex-specific indications, co-morbidities, procedural outcomes including complications and device performance parameters, alongside short- and long-term outcomes, including causes of death. Sex-specific data were obtained through a chart review of electronic health records and included demographic characteristics (age and body mass index), cardiac conditions (ejection fraction [EF], arrhythmias, valvular disease and medication use), co-morbidities, procedural details (implantation time, deployments, device position and complications) and follow-up data on device performance and clinical outcomes, including mortality. Local registration authorities (Austria National Statistics Institute) were contacted to determine patients´survival status and cause of death.

### Implantation procedure

Implantation of Micra™ LCPs was performed via the right femoral vein using specialized delivery systems. Devices were positioned in the mid-septum or apex of the right ventricle (RV). Intravenous antibiotics were administered within 60 to 120 min before implantation. For patients receiving direct oral anticoagulants, treatment was briefly interrupted before implantation and resumed on the next day after implantation. Patients receiving vitamin K antagonists had an International Normalized Ratio maintained within a low therapeutic range at the time of implantation. All procedures were conducted at a high-volume, experienced LCP implantation centre in Austria.

The study design was approved by the local ethics committee (EC number 1277/2019: amendment February 2024). The study adhered to the principles outlined in the Declaration of Helsinki.

### Statistical analysis

Categorical variables were presented as absolute numbers and percentages and for continuous variables median and interquartile range (IQR) are given. Statistical analyses included Fisher's exact test for associations between nominal or ordinal variables, Mann–Whitney U test for comparisons of continuous variables between two independent groups, Kaplan–Meier survival analysis and multivariable logistic regression models. All statistical calculations were performed using R statistical software (version 4.4.2).

## Results

### Study population and co-morbidities

Between December 2013 and October 2022, 378 LCPs were implanted in 127 women (33.6%) and 251 men (66.4%). Median age did not differ between sexes (women: 81.4 vs men: 80.5 years, *p* = *0.46*). Atrial fibrillation or flutter (AF) was more prevalent in men (75.3%) than in women (67.7%, *p* = *0.006*). Among AF subtypes, paroxysmal AF occurred more frequently in women (22.1% vs 12.0%), whereas persistent (women: 9.5% vs men: 16.3%) and permanent AF (women: 36.2% vs men: 47.0%) were more common in men. The use of non-vitamin K anticoagulants and vitamin K antagonists showed no sex-related difference (women: 68.5% vs men: 72.1%, *p* = *0.43*). More women were treated with beta-blockers (women: 48.8% vs men: 27.5%, *p* < *0.001*) and digitalis (women: 8.7% vs men: 2.8%, *p* = *0.019*). No sex-related differences were observed for co-morbidities, such as arterial hypertension, diabetes, chronic kidney disease, hypercholesterinemia, cerebrovascular disease, stroke or transient ischemic attack, peripheral artery disease and chronic obstructive pulmonary disease. Men reported coronary artery disease (40.6% vs 21.3%, *p* < *0.001)* and a history of myocardial infarction (12.4% vs 2.4%, *p* < *0.001*) more frequently than women. Median EF did not differ between sexes (women EF: 60% vs men EF: 60%). Moderate to severe valvular heart disease was more common in women (52.4% vs 40.2%, *p* = *0.03*), with numerically more transcatheter and surgical aortic valve replacements observed in women (29.9% vs 19.5%, *p* = *0.08*). Malignant diseases were reported more frequently in men (20.7% vs 7.9%, *p* = *0.001*). The baseline characteristics of the study population are summarised in Table 1.

**Table 1:** Sex-specific baseline characteristics of the study population (*n* = 378)

Parameter	Women	Men	*p-values*
	Median (IQR) or count (%)	Median (IQR) or count (%)	
	127 (33.6)	251 (66.4)	
Age (years)	81.4 (76.7–85.3)	80.5 (76.1–84.7)	*0.46*
Height (cm)	162 (158–166)	173 (170–179)	< *0.001*
Weight (kg)	68 (60–76.5)	80 (72–88.25)	< *0.001*
BMI (kg/m^2^)	25.8 (23.5–29.0)	26.1 (24.2–29.1)	*0.28*
Atrial fibrillation/flutter			*0.006*
- No AF	41 (32.3)	62 (24.7)	
- Paroxysmal AF	28 (22.1)	30 (12.0)	
- Persistent AF	12 (9.5)	41 (16.3)	
- permanent AF	46 (36.2)	118 (47.0)	
Arterial hypertension	104 (81.9)	184 (73.3)	*0.07*
Diabetes			*0.76*
- No diabetes	112 (88.2)	215 (85.7)	
- Diabetes type I	0 (0)	1 (0.4)	
- Diabetes type II	15 (11.8)	35 (13.9)	
Chronic kidney disease			*0.17*
- Grade 1	5 (4.0)	22 (8.9)	
- Grade 2	46 (36.5)	107 (43.0)	
- Grade 3	67 (53.2)	104 (41.8)	
- Grade 4	7 (5.6)	14 (5.6)	
- Grade 5	1 (0.8)	2 (0.8)	
Hypercholesterinemia	72 (59.5)	128 (52.2)	*0.09*
Coronary artery disease	27 (21.3)	102 (40.6%)	< *0.001*
Myocardial infarction Hx/o	3 (2.4)	31 (12.4)	< *0.001*
EF (%)	60 (56.25–65.0)	60 (55.0–63.0)	*0.04*
TAPSE (mm)	18.5 (17.0–22.75)	19 (17.0–22.0)	*0.95*
SPAP (mmHg)	40 (36.88–55.0)	42.5 (35.0–51.38)	*0.84*
Moderate to severe VHD	66 (52.4)	101 (40.2)	*0.03*
Aortic valve replacement			*0.08*
- TAVR before LCP	32 (25.2)	37 (14.7)	
- TAVR after LCP	2 (1.6)	6 (2.4)	
- SAVR	4 (3.2)	6 (2.4)	
Cerebrovascular disease	14 (11.0)	38 (15.1)	*0.34*
Stroke/TIA	18 (14.2)	34 (13.5)	*0.95*
Peripheral artery disease	9 (7.1)	23 (9.2)	*0.56*
COPD	10 (7.9)	23 (9.2)	*0.85*
Neoplasia	10 (7.9)	52 (20.7)	*0.001*
NOAC/OAC			*0.43*
- No NOAC or OAC	40 (31.5)	68 (27.1)	
- Apixaban	27 (21.3)	60 (23.9)	
- Dabigatran	9 (7.1)	17 (6.8)	
- Edoxaban	3 (2.4)	18 (7.2)	
- Rivaroxaban	17 (13.4)	35 (13.9)	
- Phenprocoumon	31 (24.4)	53 (21.1)	
Antiplatelet therapy			*0.85*
- No antiplatelet therapy	87 (68.5)	170 (67.7)	
- Acetylsalicylic acid	15 (11.8)	30 (12.0)	
- Clopidogrel	9 (7.1)	24 (9.6)	
- Both	16 (12.6)	27 (10.8)	
RAAS inhibitors			*0.46*
- No RAAS inhibitors	41 (32.3)	93 (37.1)	
- ACE-inhibitors	43 (33.9)	76 (30.3)	
- ARB	38 (29.9)	73 (29.1)	
- Renin inhibitors	4 (3.2)	3 (1.2)	
- MRA	1 (0.8)	6 (2.4)	
Beta-blockers	62 (48.8)	69 (27.5)	< *0.001*
Digitalis	11 (8.7)	7 (2.8)	*0.019*
Antiarrhythmic drugs			< *0.001*
- No AAD	94 (74.0)	223 (88.8)	
- Class I	1 (0.8)	2 (0.8)	
- Class II	13 (10.2)	13 (5.2)	
- Class III	8 (6.3)	3 (1.2)	
- Class IV	0 (0)	3 (1.2)	
NSAIDs (chronic treatment)	7 (5.5)	5 (2.0)	*0.12*
Steroids	4 (3.2)	9 (3.6)	*1*

*AAD* Anti-arrhythmic drugs, *ACE* Angiotensin-converting enzyme, *AF* Atrial fibrillation/flutter, *ARB* angiotensin receptor blocker, *COPD* Chronic obstructive pulmonary disease, *EF* Ejection fraction, *Hx/o* History of, *MRA* Mineralocorticoid receptor antagonist, *NOAC* Novel oral anticoagulant, *NSAID* Non-steroidal anti-inflammatory drug, *OAC* Oral anticoagulant, *RAAS* Renin–angiotensin–aldosterone system, *SAVR* Surgical aortic valve replacement, *TIA* Transient ischaemic attack, *TAVR* Transcatheter aortic valve replacement, *VHD* Valvular heart disease

### Indications, procedural outcomes and complications

The most frequent indications for LCP implantation were AF with slow conduction (women: 31.5% vs men: 44.6%), third-degree atrioventricular (AV) block (women: 31.5% vs men: 33.5%) and sick sinus syndrome (women: 21.3% vs men: 9.6%). Additional details are provided in Table 2.

**Table 2:** Sex-specific indications, procedural and electrical performance parameters of leadless cardiac pacemakers (*n* = 378; women: *n* = 127; men: *n* = 251)

Parameter	Women *n *= 127	Men *n* = 251	*p-values*
	Median (IQR) or count (%)	Median (IQR) or count (%)	
Indication			*0.004*
- AF with slow ventricular conduction	40 (31.5)	112 (44.6)	
- AV block II type 2	5 (4.0)	13 (5.2)	
- AV block III	40 (31.5)	84 (33.5)	
- Sick sinus syndrome	27 (21.3)	24 (9.6)	
- Pace and ablate for fast ventricular conduction AF	2 (1.6)	0 (0)	
- Bi-/intermittent trifascicular block	1 (0.8)	4 (1.6)	
- AV block I and LBBB	12 (9.5)	11 (4.4)	
- AV block I and RBBB	0 (0)	3 (1.2)	
Type of Micra LCP			*0.02*
- Micra VR	109 (85.8)	224 (89.2)	
- Micra AV	18 (14.2)	27 (10.8)	
Duration of implantation (min)	40 (30–54.5)	40 (30–55)	*0.69*
Fluoroscopy time (min)	5 (3.4–7.3)	4.7 (3.4–7.2)	*0.67*
Deployments of LCP at implantation	1 (1–2)	1 (1–2)	*0.55*
Position of LCP			*0.15*
- Apical	34 (26.8)	50 (19.9)	
- Septal	93 (73.2)	201 (80.1)	
Length of hospital stay (days)			
- Total	8 (5–12.5)	6 (4–10)	*0.001*
- After LCP implantation	3 (2–6)	2 (1–4)	< *0.001*
Battery voltage (V)			
- 3 months	3.13 (3.1–3.16)	3.12 (3.08–3.15)	
- 1 year	3.04 (3.03–3.05)	3.03 (3.02–3.04)	
- 2 years	3.02 (3.01–3.03)	3.02 (3.0–3.02)	
- 3 years	3.02 (2.99–3.02)	3.0 (2.99–3.02)	
- 4 years	2.99 (2.99–3.01)	2.99 (2.98–3.0)	
- 5 years	2.99 (2.97–3.0)	2.99 (2.97–2.99)	
- 6 years	2.99 (2.97–2.99)	2.97 (2.95–2.98)	
- 7 years	2.97 (2.95–2.99)	(2.94–2.97)	
- 8 years	(2.87–2.98)	(2.93)	
Pacing threshold (V/0.24ms)			
- At implantation	0.38 (0.38–0.5)	0.5 (0.38–0.63)	
- 3 months	0.5 (0.38–0.5)	0.38 (0.38–0.5)	
- 1 year	0.5 (0.38–0.63)	0.5 (0.38–0.63)	
- 2 years	0.5 (0.38–0.63)	0.5 (0.38–0.63)	
- 3 years	0.63 (0.5–0.63)	0.5 (0.38–0.63)	
- 4 years	0.5 (0.5–0.63)	0.5 (0.41–0.63)	
- 5 years	0.5 (0.5–0.72)	0.5 (0.38–0.63)	
- 6 years	0.5 (0.44–0.63)	0.5 (0.44–0.69)	
- 7 years	0.56 (0.5–0.63)	(0.38—0.5)	
- 8 years	(0.5–1.0)	(0.38)	
Sensing (mV)			
- At implantation	9.1 (6.8–13.0)	9.7 (6.9–12.8)	
- 3 months	14.3 (10.2–18.7)	12.9 (9.2–17.0)	
- 1 year	15.3 (10.9–20.0)	13.2 (9.4–17.7)	
- 2 years	15.1 (10.3–19.8)	15.3 (9.7–19.8)	
- 3 years	14.8 (11.2–20.0)	13.5 (10.0–18.7)	
- 4 years	13.4 (11.3–18.7)	14.7 (9.2–19.6)	
- 5 years	15.1 (11.7–20.0)	15.6 (11.3–19.6)	
- 6 years	13.2 (10.9–17.4)	13.1 (8.5–16.2)	
- 7 years	13.6 (9.1–20.0)	(12.0–15.4)	
- 8 years	(7.2–20.0)	(10.6)	
Impedance (Ohm)			
- At implantation	750 (640–985)	755 (640–888)	
- 3 months	605 (533–738)	570 (510–660)	
- 1 year	590 (515–690)	535 (490–600)	
- 2 years	570 (490–620)	525 (480–600)	
- 3 years	530 (480–590)	515 (470–570)	
- 4 years	515 (468–598)	520 (470–583)	
- 5 years	520 (460–560)	530 (480–568)	
- 6 years	460 (460–560)	445 (415–500)	
- 7 years	540 (465–580)	(470–640)	
- 8 years	(480–560)	(480)	
Pacing percentage			
- 3 months	33 (2–86)	58 (8–96)	
- 1 year	28 (3–89)	70 (16–95)	
- 2 years	30 (6–88)	75 (10–97)	
- 3 years	45 (7–95)	91 (21–97)	
- 4 years	56 (33–95)	86 (14–97)	
- 5 years	61 (30–97)	95 (19–99)	
- 6 years	28 (7–54)	89 (25–98)	
- 7 years	65 (9–91)	(99–100)	
- 8 years	(13–98)	(98)	
CIED extraction	19 (15.0)	27 (10.7)	*0.25*

*AF* Atrial fibrillation/flutter, *AV* Atrioventricular, *CIED* Cardiac implantable electronic device, *LBBB* Left bundle branch block, *LCP* Leadless cardiac pacemaker, *RBBB* Right bundle branch block

A total of 109 women (85.8%) and 224 men (89.2%) received a Micra VR™ LCP, whereas the remaining patients received a Micra AV™ LCP. The median duration of implantation (40 min in women and 40 min in men, *p* = *0.69*) and fluoroscopy time (5 min in women and 4.73 min in men, *p* = *0.67*) did not differ between the sexes. LCPs were implanted in the septal position of the RV in 73.2% of women and 80.1% of men, whereas the remaining LCPs were positioned apically in the RV. The median number of deployments was 1 (IQR 1–2) in both groups.

Overall complication rates during the index hospital stay were 3.9% in women (*n* = 5) and 1.6% in men (*n* = 4, *p* = *0.18*), with more events occurring during LCP implantation in women (*n* = 4, 3.1%) compared to men (*n* = 1, 0.4%) and slightly fewer complications during index hospitalisation (women: *n* = 1, 0.8% vs men: *n* = 3, 1.2%). Two women required pericardiocentesis after transvenous PM extraction and concomitant LCP implantation, and one woman with pericardial effusion was managed conservatively without pericardiocentesis. One woman experienced an unsuccessful LCP implantation; a second procedure performed one week later resulted in successful implantation. A groin hematoma with hypovolemic shock occurred in one man. During the index hospitalisation, one stroke occurred in a woman and another in a man. No LCP dislodgement events were reported in either sex.

Median total hospital stay was longer in women (eight days) compared to men (six days) (*p* = *0.001)*. Post-implantation hospital stay was three days in women and two days in men (*p* < *0.001*).

### Electrical device performance parameters

**Fig. 1:**
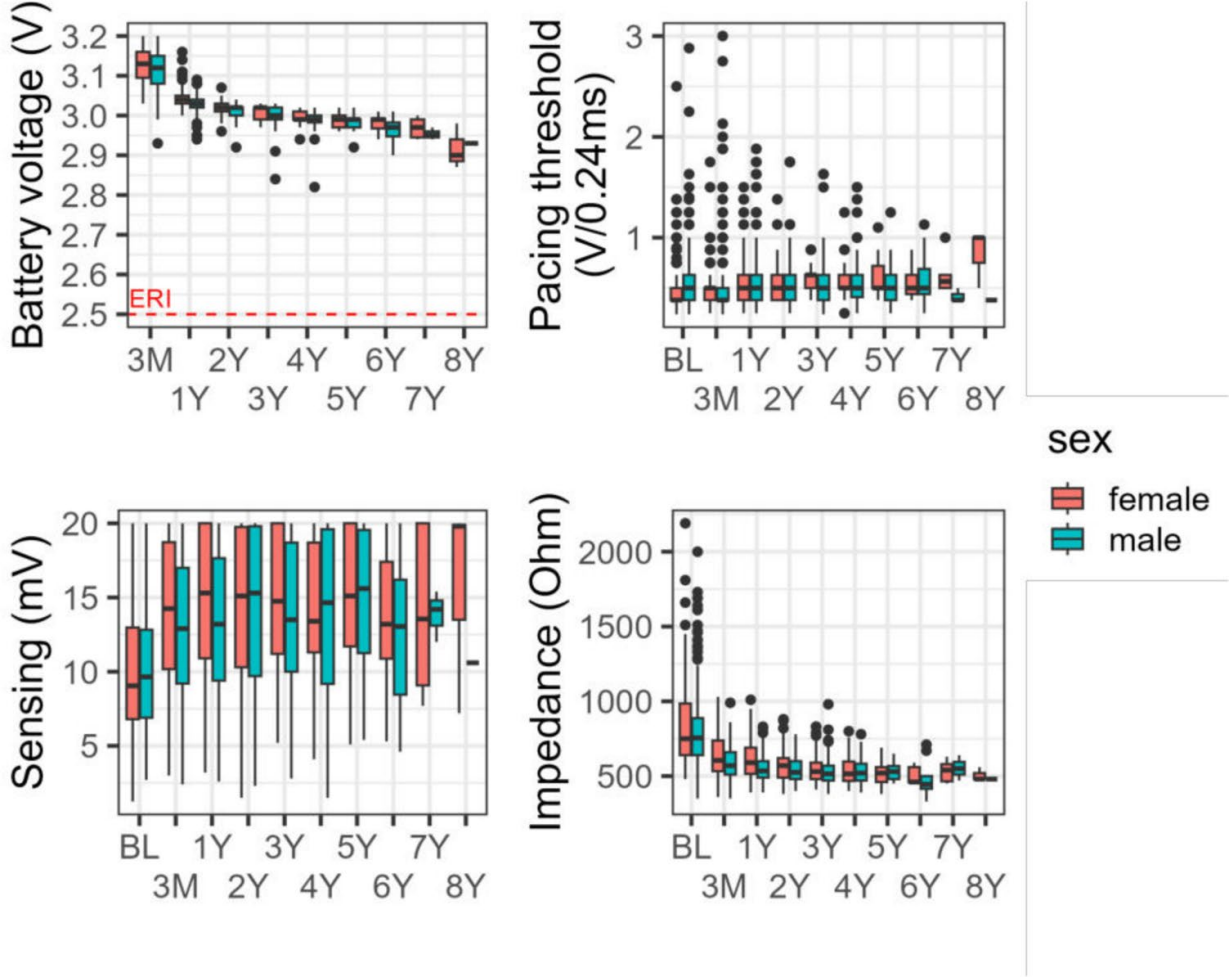
Sex-specific differences in Micra™ LCP performance parameters, including battery voltage (V), pacing threshold (V/0,24ms), sensing amplitude (mV) and impedance (Ohm). ERI = elective replacement indicator; LCP = leadless cardiac pacemaker

**Fig. 2:**
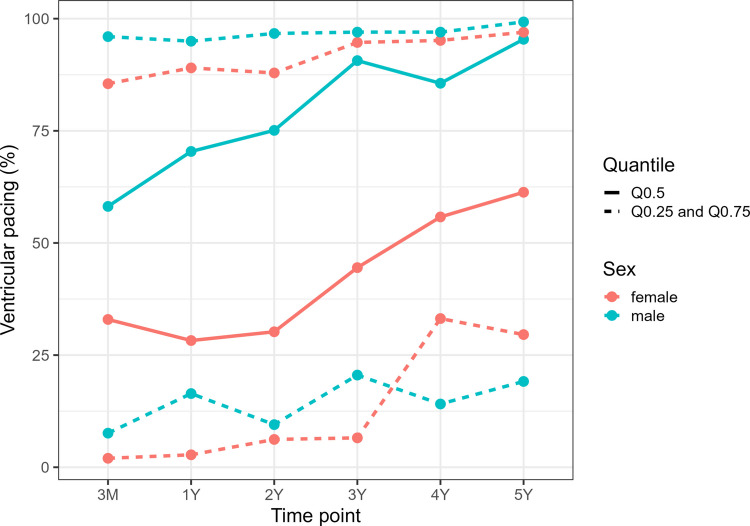
Quantile line plot of ventricular pacing (%) during follow-up.Red lines represent quartiles for female patients (solid: Q0.50; dashed: Q0.25 and Q0.75), and blue lines represent quartiles for male patients

During a median follow-up of 41 months (IQR 22–65 months), pacing thresholds remained stable, sensing values increased and impedance values decreased after LCP implantation. No clinically relevant differences emerged between women and men in electrical LCP performance parameters, including battery voltage, pacing threshold, sensing and impedance (Fig. 1). However, the pacing percentage was higher in men and increased over time in both groups (Fig. 2).

Three Micra™ LCPs were explanted in three men for the following reasons: one device, initially implanted as an interim PM in a patient with a PM-dependent third-degree AV-block following device extraction for lead endocarditis, was successfully upgraded to a CRT-D within one month. Another device was explanted due to progressively worsening sensing values, and a third due to an increased pacing threshold. Both of these patients received a transvenous PM within one month of LCP implantation.

### Short- and long-term outcomes

In-hospital mortality rates were similar between sexes, with 0.8% in women (*n* = 1) and 0.8% in men (*n* = 2). One man (84 years) experienced an acute myocardial infarction with cardiogenic shock one day after LCP implantation. Another man (87 years) died from a pulmonary abscess 18 days after device extraction and concomitant LCP implantation for lead endocarditis. One woman (66 years) died from septic shock 21 days after device extraction for lead endocarditis.

**Fig. 3:**
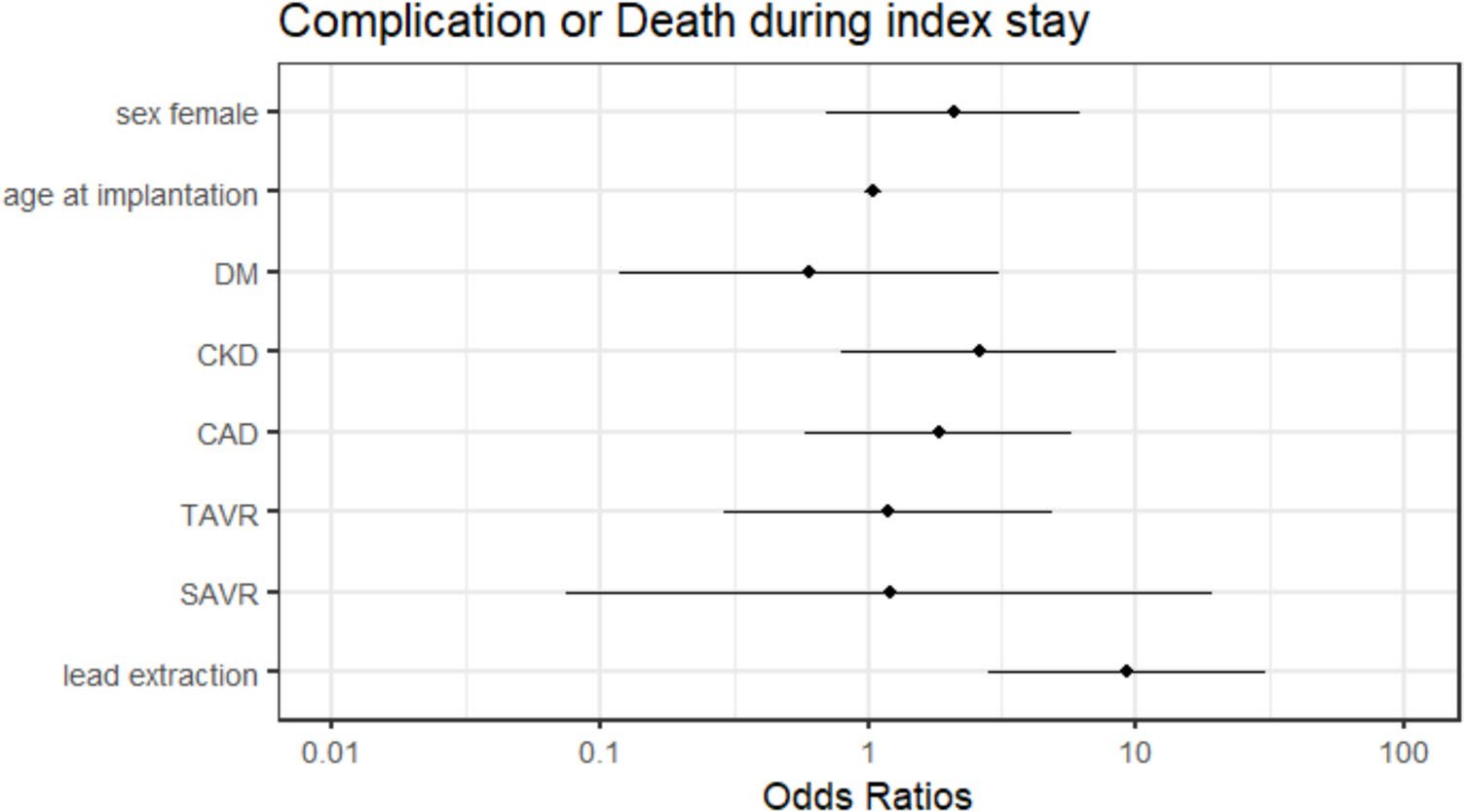
Forrest plot of penalized-likelihood logistic regression model that shows the probability of complications or death during index stay taking into account the variables sex, age at implantation, diabetes, chronic kidney disease, coronary artery disease, history of transcatheter or surgical aortic valve replacement, and concomitant transvenous lead extraction. CAD = coronary artery disease; CKD = chronic kidney disease; DM = diabetes; SAVR = surgical aortic valve replacement; TAVR = transcatheter aortivc valve replacement

A multivariable logistic regression analysis revealed an odds ratios (OR) of 2.090 for women (CI 0.627–7.197, *p* = *0.226*), 1.047 for age at implantation (CI 0.969–1.151, *p* = *0.272*), 0.604 for diabetes (CI 0.062–2.893, *p* = *0.563*), 2.595 for chronic kidney disease (CI 0.756–11.232, *p* = *0.134*), 1.820 for coronary artery disease (CI 0.485–6.440, *p* = *0.356*), 1.187 for transcatheter (CI 0.209–4.985, *p* = *0.827*) and 1.200 for surgical aortic valve replacement (CI 0.009–11.857, *p* = *0.906*) and 9.153 for concomitant transvenous lead extraction (CI 2.457–34.675, *p* = *0.001*) either to suffer complications or death during index stay (Fig. 3 and Table 3).

**Table 3:** Penalized-likelihood logistic regression model that shows the probability of complications or death during index stay taking into account the variables sex, age at implantation, diabetes, chronic kidney disease, coronary artery disease, history of transcatheter or surgical aortic valve replacement, and concomitant transvenous lead extraction

	Estimate	Std. Error	Odds Ratio	CI-L (OR)	CI-U (OR)	*p-values*
(Intercept)	−4.882	0.768	0.008	0.001	0.032	*0.000*
Sex female	0.737	0.554	2.090	0.627	7.197	*0.226*
Age at implantation	0.046	0.038	1.047	0.969	1.151	*0.272*
Diabetes	−0.505	0.830	0.604	0.062	2.893	*0.563*
Chronic kidney disease	0.954	0.604	2.595	0.756	11.232	*0.134*
Coronary artery disease	0.604	0.583	1.829	0.485	6.440	*0.356*
TAVR	0.171	0.721	1.187	0.209	4.985	*0.827*
SAVR	0.183	1.410	1.200	0.009	11.857	*0.906*
Lead extraction	2.214	0.606	9.153	2.457	34.675	*0.001*

*CI-L (OR)* Confidence interval lower limit (odds ratio), *CI-U (OR)* Confidence interval upper limit (odds ratio), *SAVR* Surgical aortic valve replacement, *Std.* Standard, *TAVR* Transcatheter aortic valve replacement

**Fig. 4:**
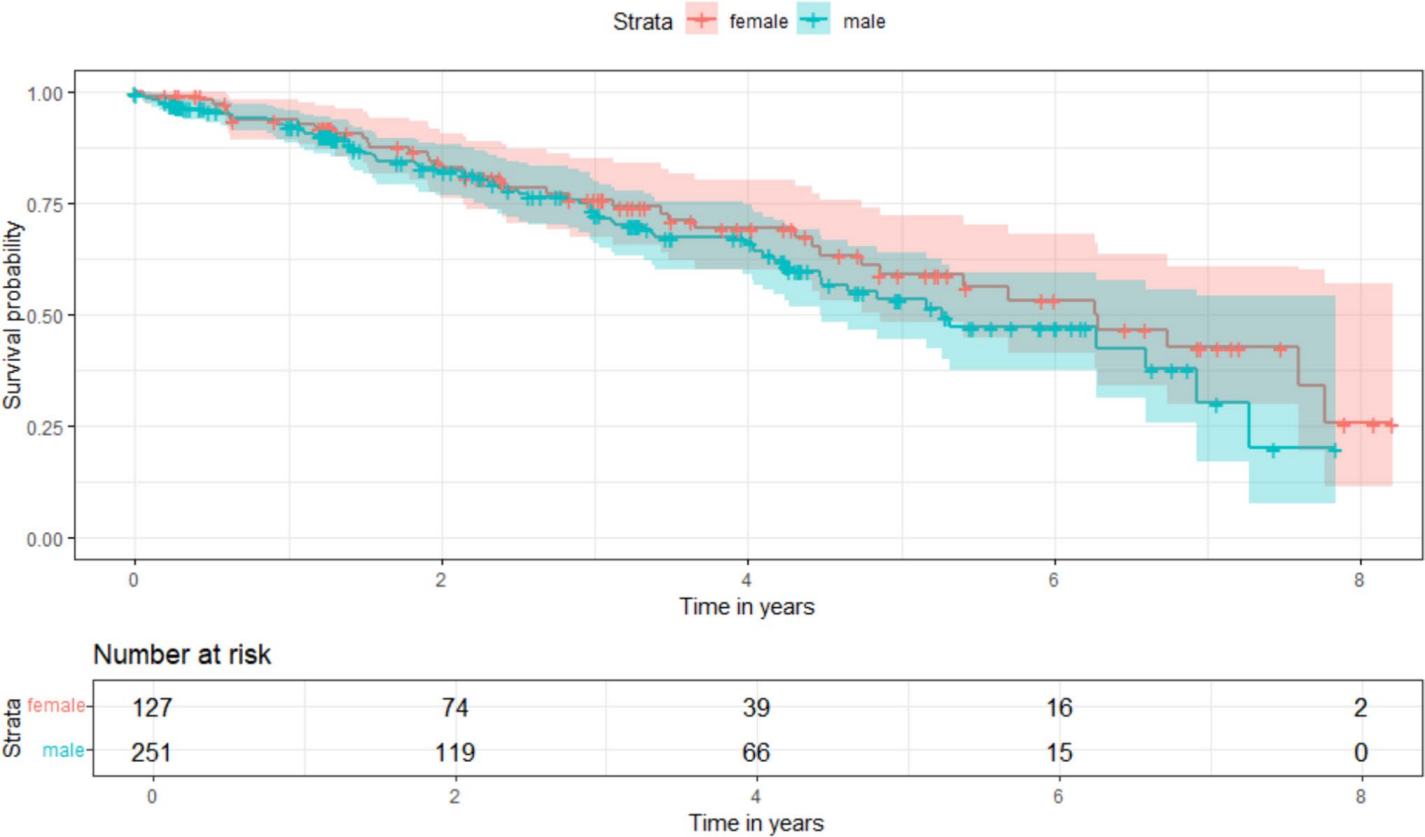
Kaplan–Meier survival curves for female (red) and male (blue) patients from the date of implantation until death or last follow-up. The table below displays the number of patients at risk at each time point

During follow-up, 39 women (30.7%) and 69 men (27.5%) died (*p* = *0.28*) (Fig. 4). Causes of death were available in 75 of the deceased women and men (69.5%). The most frequent causes of death in women were cardiovascular (20.5%), renal (12.8%) and infectious diseases (10.3%), whereas men mainly died from cardiovascular (37.6%), malignant (16.9%) and neurological diseases (5.8%). Further causes of death are presented in Table 4.

**Table 4:** Sex-specific short- and long-term outcomes including procedure-related complications during index stay, LCP explantations, mortality and causes of death in patients with leadless cardiac pacemakers (*n* = 378)

Parameter	Women *n *= 127	Men *n* = 251	*p-values*
	Count (%)	Count (%)	
Complications during index stay	5 (3.9)	4 (1.6)	*0.18*
- During LCP implantation procedure	4 (3.1)	1 (0.4)	
- After LCP implantation during index stay	1 (0.8)	3 (1.2)	
Types of complications during index stay			
- Pericardial effusion requiring pericardiocentesis	2 (1.6)	0 (0)	
- Pericardial effusion without requiring pericardiocentesis	1 (0.8)	0 (0)	
- Unsuccessful LCP implantation	1 (0.8)	0 (0)	
- Groin hematoma with hypovolemic shock	0 (0)	1 (0.4)	
- Pseudoaneurysm	0 (0)	2 (0.8)	
- Stroke	1 (0.8)	1 (0.4)	
- LCP dislodgement	0 (0)	0 (0)	
LCP explantations	0 (0)	3 (1.2)	*0.55*
In-hospital mortality during index stay	1 (0.8)	2 (0.8)	*0.96*
- septic shock after device extraction for lead endocarditis	1 (0.8)	0 (0)	
- pulmonary abscess after device extraction for lead endocarditis	0 (0)	1 (0.4)	
- acute myocardial infarction complicated by cardiogenic shock	0 (0)	1 (0.4)	
All-cause mortality during index stay and follow up	39 (30.7)	69 (27.5)	*0.28*
Causes of death during index stay and follow up			
- Cardiovascular	8 (20.5)	26 (37.6)	
- Renal	5 (12.8)	2 (2.7)	
- Infectious	4 (10.3)	3 (4.3)	
- Neurological	3 (7.7)	4 (5.8)	
- Marasmus	2 (5.1)	0 (0)	
- Malignant	1 (2.6)	11 (16.9)	
- Diabetes	1 (2.6)	2 (2.7)	
- Pulmonary	0 (0)	1 (1.3)	
- Hepatological	0 (0)	1 (1.3)	
- Trauma	0 (0)	1 (1.3)	
- missing data (unkown cause of death)	15 (38.4)	18 (26.1)	

## Discussion

The main findings of this registry study are as follows: Firstly, a less frequent use of LCPs in women (33.6%) compared to men (66.4%) was observed. Secondly, indications for LCP therapy were different between women and men (AF with slow conduction 31.5% vs 44.6%, third-degree AV block 31.5% vs 33.5%, sick sinus syndrome 21.3% vs 9.6%). Procedural results did not differ between both sexes. However, women experienced numerically more complications (women: 3.9% vs men: 1.6%), although no difference between sexes was found (*p* = *0.18*). Thirdly, LCP performance parameters, including pacing threshold, sensing, impedance and battery voltage, did not differ between sexes. An increasing ventricular pacing percentage during follow-up was observed in both sexes, with a higher percentage in men. Finally, in-hospital mortality (women: 0.8% vs men: 0.8%, *p* = *0.96*) remained similar, and all-cause mortality during follow-up (women: 30.7% vs men: 27.5%) was higher in women compared to men (*p* = *0.28*).

Several studies have reported a lower percentage of cardiac implantable electronic device (CIED) implantations and higher rates of major adverse procedural complications in women, as well as comparable in-hospital all-cause mortality for both sexes and better long-term survival in women [2, 4, 9–12]. This observation was also reported for LCP in the 5-year follow-up of the Micra transcatheter pacing system post-approval registry, which included 38.8% of women [13]. To date, three studies have been published comparing sex-specific differences in patients undergoing LCP implantation [5, 6, 14]. Baseline data, including age and co-morbidities, in our study cohort were comparable to those studies. The largest and most recent United States database study by Khan et al. reported an lower utilisation of LCPs in women (44.7%), a finding consistent with our study (33.6% in women) [6]. The reasons for this less frequent use of LCP in women remain unclear. Well-documented sex differences in electrocardiography exist, such as longer QTc intervals in women and higher T-wave amplitudes and ST angles, which are mainly attributed to the influence of sex hormones on cardiac repolarisation [15]. Women are more likely to develop sinus node disease, whereas men are more likely to present with AV block [16]. The most frequent indications for LCP implantation differed between sexes in our study were AF with slow conduction (31.5% vs 44.6%), third-degree AV block (31.5% vs 33.5%) and sick sinus syndrome (21.3% vs 9.6% in women vs men, respectively). According to current ESC guidelines and a national expert consensus document of the Austrian Society of Cardiology, LCP may be considered in patient with SSS and AV block with a low anticipated ventricular pacing rate (< 1–5% of beats) and with increased anticipated ventricular pacing rate (> 1–5% of beats) under certain circumstances (e.g. older age and co-morbidities). Micra AV™ LCP with VDD pacing may be a therapeutic option with mean percentage of AV synchrony of 89.2% (median: 94.3%) [17].

Sex differences in cardiac arrhythmia are well summarized in a consensus document published by the European Heart Rhythm Association [18]. With respect to PM therapy, women are older at implantation [2, 19–21]. Remarkably, studies demonstrated a longer survival of women compared to men despite an older age at implantation [19, 22]. Women require PMs more likely for SSS [2, 19]. The most frequent indication in men is AV block [12, 23]. A male predominance for PM therapy is reported in the literature [20, 24]. The Austrian PM registry reported 7.592 PM implantations (including transvenous PM, LCP and CRT-P) in 2024 and 5.625 of these were first implantations (2.288 women, 40.7% and 3.337 men, 59,3%) [25]. However, an older study from the Netherlands demonstrated no sex differences with respect to choice of pacing systems [26]. The decision was more affected by age. Newer cardiac PM and techniques, such as conduction system pacing, allow an individual approach considering aetiology, age and comorbidities. Recent study results demonstrated similar clinical outcomes for women and men undergoing left bundle branch area pacing for cardiac resynchronization therapy [27]. Although, women reported lower quality of life after PM implantation, age seems to affect quality of life more than sex [28, 29]. An observational study assessing quality of life comparing conventional transvenous PM and LCP revealed better results in physical and mental health with less procedure-related discomfort, physical restriction and preoccupation for LCP [30]. With respect to complications, Kirkfeldt et al. demonstrated higher rates for women undergoing CIED implantations (PM, CRT-P, CRT-D and ICD) with an adjusted RR (aRR) of 1.3 (95% CI: 1.1–1.6) [31]. Other risk factors were underweight with a BMI < 18.5 (aRR 1.5; 1.1–2.3), annual volume of implantations per centre < 750 procedures (500–749: aRR 1.5; 1.2–1.8) and per operator < 50 procedures (aRR 1.9; 1.4–2.6), dual-chamber ICD (aRR 2.0; 1.4–2.7) and CRT-D (aRR 2.6; 1.9–3.4) implantations, system upgrades or lead revisions (aRR 1.3; 1.0–1.7) and emergency, out of hours procedures, respectively.

We did not observe clinically relevant sex-specific differences in electronic LCP performance parameters, including battery voltage, pacing threshold, sensing and impedance during follow-up (median: 41 months, IQR 22–65 months). This finding is consistent with results from Huang et al. and Mitacchione et al. [5, 14]. Remarkably, a sex-specific difference in the percentage of ventricular pacing was observed, with a higher proportion in men compared to women (Fig. 2), which may be explained by the differing indications for LCP therapy noted above. Remarkably, this difference did not result in a lower battery voltage in women during follow-up.

Although procedural data (duration of implantation, fluoroscopy time and deployments) did not differ between both sexes, a numerically higher rate of complications was observed in women (women: 3.9% vs men: 1.6%, *p* = *0.18*). These complications occurred more frequently during LCP implantation (women: 3.1% vs men: 0.4%). Khan et al. reported even higher rates of major complications (women: 8.6% vs men: 8.5%, *p* = *0.76*), including pericardial effusion requiring intervention and vascular complications such as AV fistula, pseudoaneurysm, access site hematoma, retroperitoneal bleeding and venous thromboembolism [6]. In this study, pericardial effusions requiring pericardiocentesis were observed only in two women (1.6%) who underwent PM extraction and concomitant LCP implantation. Khan et al. documented a 1.1% rate of pericardial effusion requiring intervention in patients undergoing LCP implantation, with an odds ratio (OR) of 2.03 for female sex (95% confidence interval [CI]: 1.62–2.55) [32]. This complication was independently associated with mortality (OR 5.66, 95% CI: 4.24–7.56). In contrast, Huang et al. and Mitacchione et al. did not observe sex-specific differences in major LCP-related implantation complications [5, 14]. Smaller heart size and vascular access are potential risk factors for a higher rate of procedural complications in women, particularly in the context of LCPs requiring intracardiac fixation and device placement. However, device-related complications are lower for LCPs compared to transvenous PMs, as recently confirmed in the 5-year follow-up of the Micra transcatheter pacing system post-approval registry [13]. The safety of the Micra™ LCP system has been well documented in several studies [33–35].

The finding of a prolonged hospitalisation in women (total: eight days; post-implantation: three days) compared to men (total: six days, *p* = *0.001*; post-implantation: two days, *p* < *0.001*) during index stay for LCP implantation is remarkable. However, as procedural parameters did not differ between sexes and complications rates were low for women (*n* = 5; 3.9%) and men (*n* = 4,;1.6%; *p* = *0.18*), we believe from a clinical perspective that social factors, such as frailty, limited availability of transition care especially for women and lack of open spaces in nursing homes, had more impact on the length of hospitalisation.

Overall, three LCP were explanted during the study period: Two devices required an upgrade to conventional transvenous PM due to progressively worsening of sensing values and increased pacing threshold within one month after LCP implantation. In one case the LCP was used as an interim PM because of lead endocarditis in a patient with a PM-dependent third-degree AV block after DDD PM extraction and recurrent infection with vegetations on the interim transvenous PM lead. Finally. a CRT-D was implanted because of severely reduced EF and the LCP was successfully explanted. Recently published studies demonstrated the feasibility and a good outcome of LCP implantation in patients undergoing CIED extraction for infection or dysfunction [36, 37]

In-hospital mortality in our study cohort was low and identical between sexes (0.8% for both men and women, *p* = *0.96*), contrasting with higher rates reported by Khan et al. (5.5% in women vs 4.5% in men, *p* < *0.01*), Huang et al. (30-day all-cause mortality 6.7% in women vs 6.8% in men, *p* = 0.99) and Mitacchione et al. (all-cause mortality 6.8% in both sexes) [5, 6, 14]. Concomitant transvenous lead extraction (OR 9.153, CI 2.457–34.675, *p* = *0.001)* was the only predictor of complications or death during index stay in a multivariable logistic regression adjusted for sex, age at implantation, diabetes, chronic kidney disease, coronary artery disease, transcatheter and surgical valve replacement, while female sex did not affect short-term outcomes (OR 2.090, CI 0.627–7.197, *p* = *0.226*) in our study population.

The all-cause mortality during long-term follow-up was 30.7% for women and 27.5% for men in our study (*p* = *0.28*). Cardiovascular disease represented the leading cause of death in both sexes (20.5% in women vs 37.7% in men), followed by renal (12.8%) and infectious (10.3%) diseases in women, and malignant (16.9%) and neurological (5.8%) diseases in men.

This study had several limitations. Firstly, the design was a single-centre retrospective observational study rather than a prospective randomised trial. A sex-based selection bias for or against LCPs could not be excluded. However, patient selection was based on the current ESC guidelines for cardiac pacing and a national expert consensus of the Austrian Society of Cardiology [1, 7, 8]. Secondly, the findings were limited to Micra™ LCP (Medtronic, Minneapolis, MN, USA). All LCP implantations were performed by a highly experienced team of interventional cardiologists at a high-volume Austrian centre for LCP implantations. Thirdly, results of clinical outcome and EF were not available in this study as these parameters were not evaluated in all patients during follow-up and a potential risk of selection bias could not be excluded. Further prospective studies are needed to confirm these observational findings and to support the development of sex-specific protocols for safe LCP implantations in both women and men.

## Conclusions

No sex-specific disparities were observed in our study cohort with respect to complications during index stay, in-hospital and all-cause mortality. Different clinical indications for LCP therapy may have contributed to a less frequent use of LCPs in women in this single-centre observational study. Electronical performance parameters did not differ between sexes at implantation and during follow-up, although men had a higher ventricular pacing percentage. In-hospital mortality remained low for both women and men. Although numerically more procedure-related complications occurred during LCP implantation in women, no difference in complication rates during the index hospitalisation was observed between the sexes. Concomitant transvenous lead extraction was the only predictor of complication or death during index stay. Further prospective studies are warranted to establish sex-specific recommendations for LCP therapy.

## Data Availability

The data that support the findings of this study are not openly available due to reasons of sensitivity and are available from the corresponding author upon reasonable request.

## Abbreviations

*AF*: Atrial fibrillation
*aRR*: adjusted risk ratio
*AV*: atrioventricular
*CI*: Confidence interval
*CIED*: Cardiac implantable electronic device
*CRT*: Cardiac resynchronization therapy
*EF*: Ejection fraction
*ESC*: European Society of Cardiology
*IQR*: Interquartile range
*LCP*: Leadless cardiac pacemaker
*OR*: Odds ratio
*PM*: Pacemaker
*RV*: Right ventricle
